# A randomized clinical trial of a theory-based fentanyl overdose education and fentanyl test strip distribution intervention to reduce rates of opioid overdose: study protocol for a randomized controlled trial

**DOI:** 10.1186/s13063-020-04898-8

**Published:** 2020-11-26

**Authors:** Brendan P. Jacka, Jacqueline E. Goldman, Jesse L. Yedinak, Edward Bernstein, Scott E. Hadland, Jane A. Buxton, Susan G. Sherman, Katie B. Biello, Brandon D. L. Marshall

**Affiliations:** 1 Department of Epidemiology, Brown University School of Public Health, Providence, Rhode Island USA; 2 Department of Medicine, Boston University School of Medicine, Boston, Massachusetts USA; 3 School of Public Health, Boston University, Boston, Massachusetts USA; 4 Grayken Center for Addiction, Boston Medical Center, Boston, Massachusetts USA; 5 Department of Pediatrics, Boston Medicine Center, Boston, Massachusetts USA; 6 Division of General Pediatrics, Department of Pediatrics, Boston University School of Medicine, Boston, Massachusetts USA; 7 School of Population and Public Health, University of British Columbia, Vancouver, British Columbia Canada; 8 British Columbia Centre for Disease Control, Vancouver, British Columbia Canada; 9 Department of Health, Behavior, and Society, Johns Hopkins Bloomberg School of Public Health, Baltimore, Maryland USA; 10 Department of Behavioral and Social Sciences, School of Public Health, Brown University, Providence, Rhode Island United States; 11 Fenway Institute, Fenway Health, Boston, Massachusetts USA

**Keywords:** Opioid overdose, Behavioral intervention, Motivational interviewing, Fentanyl test strip, Illicitly manufactured fentanyl, Overdose prevention, Information-motivation-behavioral model, Randomized controlled trial

## Abstract

**Background:**

Opioid overdose deaths involving synthetic opioids, particularly illicitly manufactured fentanyl, remain a substantial public health concern in North America. Responses to overdose events (e.g., administration of naloxone and rescue breathing) are effective at reducing mortality; however, more interventions are needed to prevent overdoses involving illicitly manufactured fentanyl. This study protocol aims to evaluate the effectiveness of a behavior change intervention that incorporates individual counseling, practical training in fentanyl test strip use, and distribution of fentanyl test strips for take-home use among people who use drugs.

**Methods:**

Residents of Rhode Island aged 18–65 years who report recent substance use (including prescription pills obtained from the street; heroin, powder cocaine, crack cocaine, methamphetamine; or any drug by injection) (*n* = 500) will be recruited through advertisements and targeted street-based outreach into a two-arm randomized clinical trial with 12 months of post-randomization follow-up. Eligible participants will be randomized (1:1) to receive either the RAPIDS intervention (i.e., fentanyl-specific overdose education, behavior change motivational interviewing (MI) sessions focused on using fentanyl test strips to reduce overdose risk, fentanyl test strip training, and distribution of fentanyl test strips for personal use) or standard overdose education as control. Participants will attend MI booster sessions (intervention) or attention-matched control sessions at 1, 2, and 3 months post-randomization. All participants will be offered naloxone at enrolment. The primary outcome is a composite measure of self-reported overdose in the previous month at 6- and/or 12-month follow-up visit. Secondary outcome measures include administratively linked data regarding fatal (post-mortem investigation) and non-fatal (hospitalization or emergency medical service utilization) overdoses.

**Discussion:**

If the RAPIDS intervention is found to be effective, its brief MI and fentanyl test strip training components could be easily incorporated into existing community-based overdose prevention programming to help reduce the rates of fentanyl-related opioid overdose.

**Trial registration:**

ClinicalTrials.gov NCT04372238. Registered on 01 May 2020

## Background

Opioid overdose morbidity and mortality remain substantial public health threats in North America, particularly in the era of highly potent novel synthetic opioid agents [1]. Mortality related to opioid overdose is also a global concern, with the greatest impact in North America, Europe, and Australasia [2]. Contamination of the street drug supply with illicitly manufactured fentanyl (IMF) and its analogs has precipitated dramatic increases in mortality in the USA and Canada, representing a period of substantially increased risk for people who use drugs (PWUD) [3]. As IMF contamination expands to illicit stimulant and other non-opioid substances, the potential number of opioid-naïve individuals at risk of opioid overdose has also increased [4, 5].

Overdose prevention efforts include medications for opioid use disorder, overdose education and naloxone distribution (OEND) programs, overdose prevention sites and supervised consumption services, and drug testing services [6–8]. Studies show that PWUD modify or adapt their drug use behavior in light of increased overdose risk associated with synthetic opioid contamination, including using smaller doses to test drug strength, maintaining consistent drug supply, consuming drugs with others present, reducing drug consumption, and having naloxone present [9, 10]. However, as contamination of the drug supply with IMF becomes more prevalent, novel tools to prevent and respond to opioid overdose events are urgently needed.

In recent years, analytic tests to detect fentanyl contamination have received substantial attention [11]. Traditional forensic testing technologies such as mass spectrometry can identify fentanyl and other synthetic analogs in drug samples, but are expensive and require specialized training and reagents [12]. Rapid and simple point of care tests have been proposed as an alternative tool for increasing access to fentanyl testing services in highly marginalized communities, and in drug-using populations without access to supervised consumption facilities, as is currently the case in the USA. Paper-based immunoassays (e.g., Rapid Response™ fentanyl test strip, BTNX Corporation, Canada) provide similar sensitivity and specificity compared to portable mass spectrometers, with low technical requirements and results within 5 min [13]. People who use drugs report interest in knowing if fentanyl is in their drugs prior to use, with high willingness and acceptability to use fentanyl test strips in the future [14, 15]. Furthermore, early studies have shown that positive fentanyl test strip outcomes result in positive drug use behavior change, and participants report a desire to perform testing prior to drug use and in private locations [16, 17]. Novel drug testing modalities that increase harm reduction self-efficacy will be paramount in assisting and supporting PWUD at risk of fentanyl overdose.

The study protocol described herein aims to test the efficacy of a novel behavior change intervention that incorporates fentanyl testing strips with a theory-driven opioid overdose risk reduction component. Guided by two health behavior frameworks, we hypothesize that combining motivational interviewing counseling techniques to increase willingness and self-efficacy to use fentanyl test strips with fentanyl test strip training and teach-back will result in enhanced overdose risk reduction practice, compared to participants receiving standard overdose education and naloxone training. We hypothesize that exposure to the Rhode Island Prescription and Illicit Drug Study (RAPIDS) intervention will reduce rates of fatal and non-fatal opioid overdose. Mediation analysis will assess the differential uptake of behavior changes in participants receiving the intervention.

## Methods/design

### Participants, interventions, and outcomes

#### Study setting

The RAPIDS Clinical Trial will be undertaken in the state of Rhode Island, a population of 1 million with sustained high rates of overdose mortality associated with any opioids (25.9 per 100,000 people in 2017) [1]. Death involving synthetic opioids other than methadone (mostly fentanyl) increased substantially both nationally and within Rhode Island between 2013 and 2018, accounting for eight in 10 fatal overdose events in the state [1]. Opioid overdose deaths in Rhode Island involving both fentanyl and other opioids appear geographically constrained, with hot spots of overdose deaths observed in a few urban centers in the state [18].

#### Eligibility criteria

Inclusion criteria for the study are (a) 18 to 65 years of age; (b) reside in Rhode Island; (c) able to complete interviews in English; (d) able to provide informed consent, and (e) report past 30-day use of heroin, illicit stimulants (e.g., powder cocaine, crack cocaine, methamphetamine), counterfeit prescription pills, or any drug by injection, regardless of treatment status. Exclusion criteria are (a) refusal of consent to participate; (b) unable to provide informed consent due to altered mental status; (c) unable to adequately hear and/or comprehend the consent process; and (d) use of prescription medications exclusively obtained from a physician or diverted from someone else’s prescription.

#### Patient and public involvement

No patient involved.

#### Interventions

**Fig. 1:**
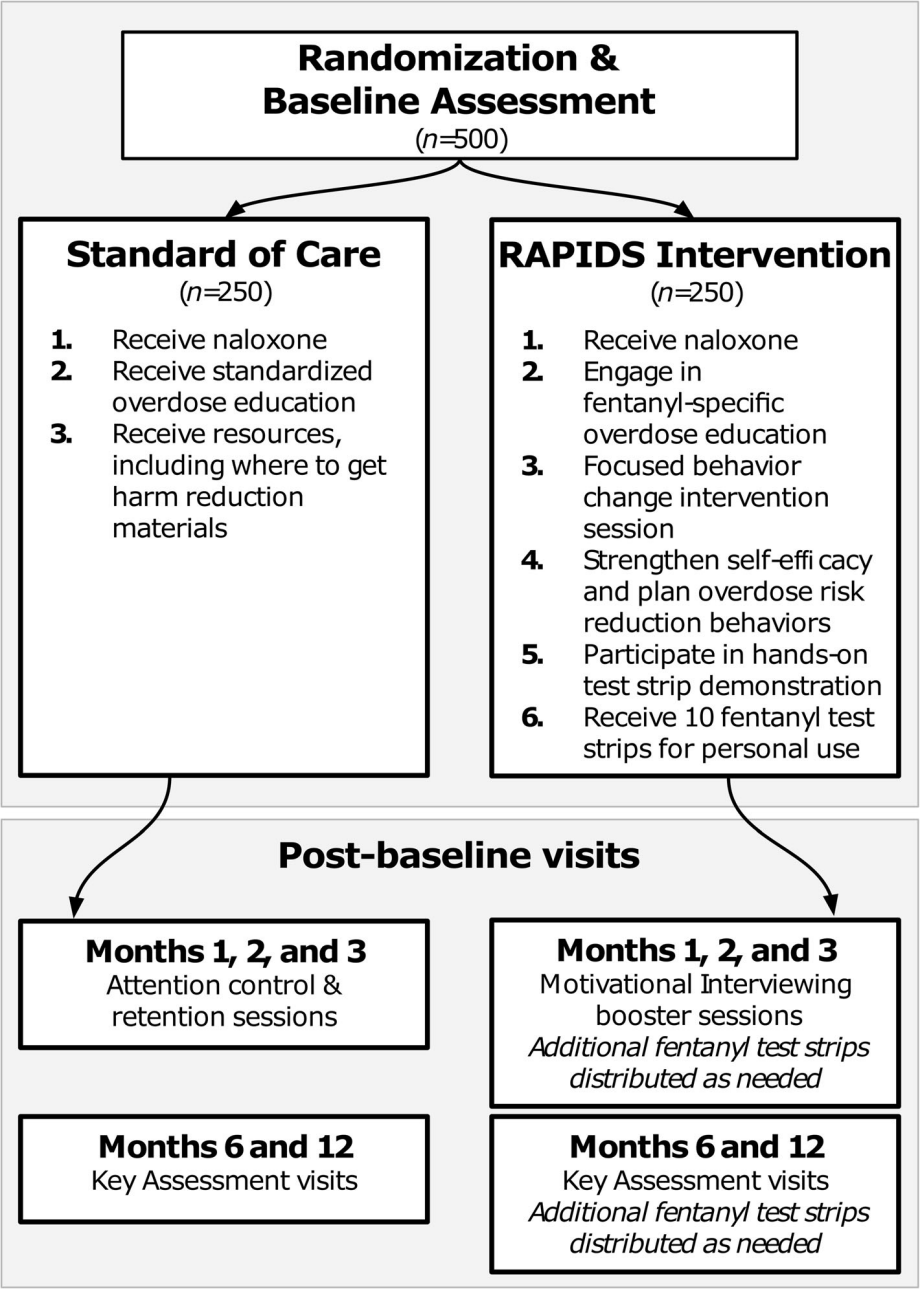
Study plan schematic of the RAPIDS Clinical Trial for the prevention of opioid overdose among people who use drugs

All participants will be randomized to receive either the RAPIDS intervention or a control condition (usual care). An overview of the study design is shown in Fig. 1; intervention components are described below.

##### Usual care

Participants in the control arm will receive standardized OEND training, as currently occurs in Rhode Island for people who are at risk for an overdose [19]. Based on common components of many community-based overdose prevention interventions, the OEND training includes information on overdose recognition and response, including an educational video on recognizing overdose symptoms, and basic overdose response (e.g., performing rescue breaths, administering naloxone, and calling for medical assistance). All participants will be offered a naloxone kit and education material at baseline, and referrals to community locations for no-cost or low-cost naloxone access will be provided at subsequent study visits.

##### Motivational interviewing-based brief intervention (RAPIDS intervention)

**Fig. 2:**
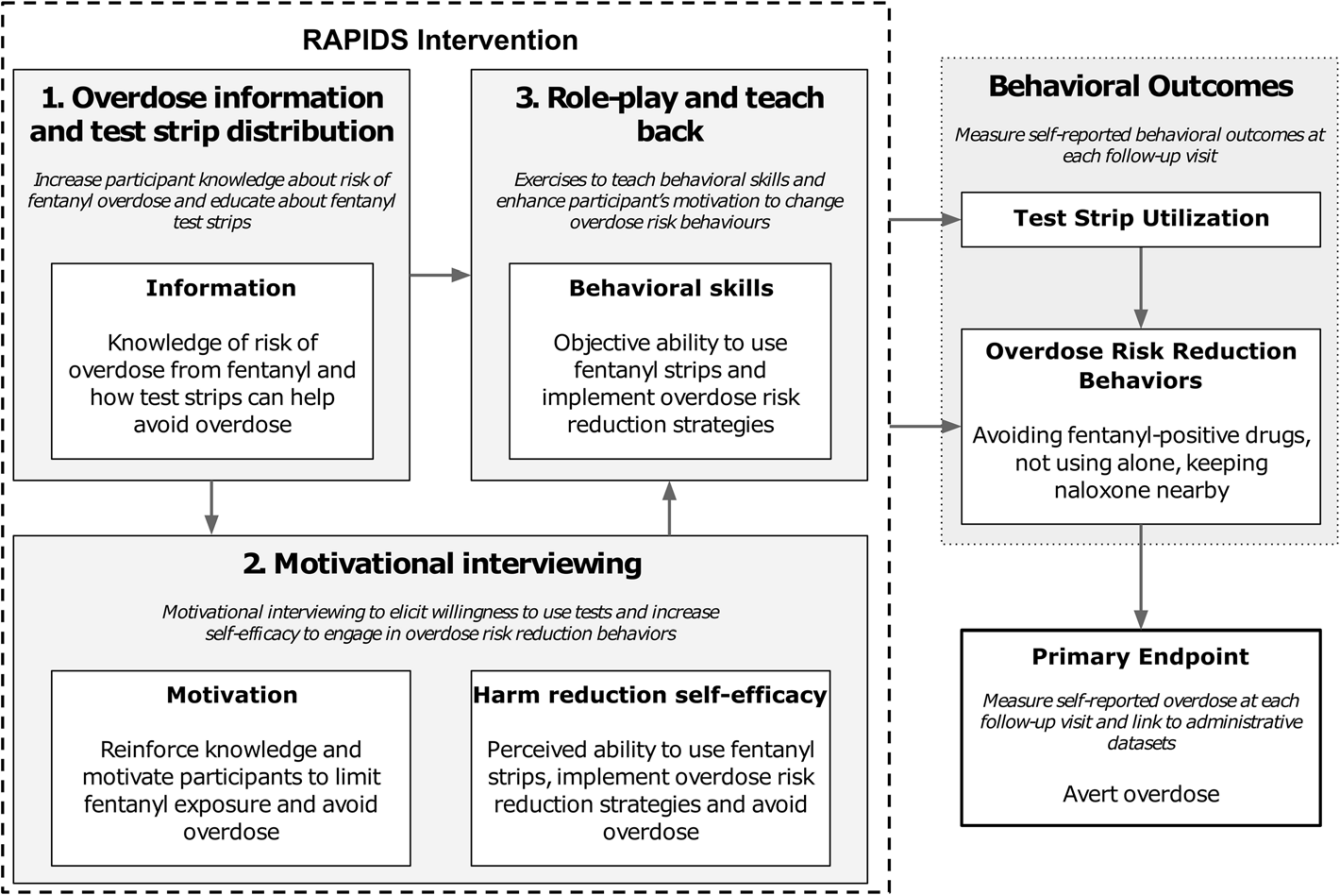
Conceptual model of the RAPIDS intervention for the prevention of opioid overdose among people who use drugs

Participants in the RAPIDS intervention arm will receive (1) a brief (<15 min) overdose education and behavior change counseling session using motivational interviewing; (2) fentanyl test strip training, role-play, and teach-back; and (3) training videos at the baseline study visit (Fig. 1). The intervention is informed by the information-motivation-behavioral skills (IMB) model of behavior change, which hypothesizes that if a person possesses the information, motivation, and behavioral skills to act, there is an increased likelihood that they will fulfill and maintain the desired behaviors [20]. The IMB model has been studied extensively and has empirical support as a framework for understanding both drug and sexual risk behaviors [21–23]. Participants will receive ten Rapid Response™ fentanyl test strips for personal use at the end of the session (FYL-1S48-100; detection cut-off, 20 ng/ml) [13]. Motivational interviewing “booster” sessions will occur at 1, 2, and 3 months post-randomization, with additional test strips provided during the booster session on an as needed basis. The goals of this intervention are to provide participants with (1) information about the risks of IMF exposure and how to reduce their risk of overdose, including naloxone training; (2) motivational interviewing to assess participants’ willingness and enhance self-efficacy for engaging in overdose risk reduction behaviors; (3) an instructional video and hands-on training to increase behavioral skills in fentanyl rapid test strip use and interpretation, and opportunities to practice using the test strips; and (4) provision of a supply of test strips for personal use. A conceptual framework outlining intervention components (informed by the IMB model) is shown in Fig. 2. Implementation of the intervention will be undertaken by research staff with a history of working in community settings to enable future scalability in resource-constrained settings such as needle-syringe programs, emergency departments, and health clinics [24–26].

During the brief intervention (approximately 10–15-min duration), interventionists will explore the participants’ familiarity with basic overdose recognition and response. Interventionists will discuss characteristics of fentanyl overdose and appropriate response with participants (e.g., rapidity of fentanyl overdose; naloxone as opioid overdose reversal) before checking with participants for understanding of participants and assessment of response. As part of fentanyl test strip training, participants will review the control arm educational video on overdose recognition and response, followed by an instructional video on the use and interpretation of fentanyl test strips. The fentanyl test strip training also includes practice test strip use and role-play risk reduction behavior given a positive or negative test strip result. Motivational interviewing processes that incorporate engaging, focusing, evoking, and planning [27] with participants will guide the direction of subsequent conversations. Using the core skills of motivational interviewing (i.e., open-ended questions, offering affirmations, providing reflections, and summarizing) [28], participants and interventionists will explore the participants’ responses to the information component of the intervention. Current risk reduction behaviors endorsed by participants will be affirmed by interventionists, and possible ambivalence about reducing overdose risk will be reflected upon. Taken together, these elements will provide a foundation for the interventionist to elicit change talk from a participant as they increase motivation, strengthen self-efficacy, and plan overdose risk reduction behaviors.

In light of the high potency of fentanyl and difficulty differentiating IMF from heroin and many illicit stimulants [11], the behavioral skills building component of the intervention will focus on overdose risk reduction behaviors considered most effective in the era of fentanyl [9, 10]. Interventionists will discuss with participants to build skills related to (1) use of drugs with someone else present who could intervene and take action in the event of an overdose; (2) obtaining naloxone and having someone present to administer if necessary; (3) calling 911 immediately if an overdose occurs; and (4) using fentanyl test strips prior to drug consumption.

#### Outcomes

The primary outcome will be a composite measure of self-reported overdose (defined as a “negative reaction from using too much drugs,” as operationalized previously [29, 30]) in the previous month at the 6- and/or 12-month follow-up visit. This sensitive definition is especially prudent in the era of IMF, where atypical presentations of opioid overdose may include chest wall rigidity, nausea, and vomiting [31]. Self-reported overdose (compared to administrative data linkage) was selected as the primary outcome measure since a minority of participants in a pilot study reported attending a hospital at their last overdose [32]. Furthermore, one of our target behaviors of the intervention includes calling 911 and seeking immediate medical attention if an overdose occurs, possibly leading to an increased rate of presentation at emergency departments for overdose events in the intervention arm. Nonetheless, exploratory analysis will utilize deterministic linkage of administrative health data systems in Rhode Island to identify fatal (through medical examiner reports) and non-fatal (through hospitalization or emergency medical service utilization) overdose events among participants.

#### Participant timeline

The trial consists of a 12-week behavioral intervention phase with a 40-week follow-up phase. The total trial period for participants will be 12 months. As shown in Table 1, measurements will be undertaken at six time points in each arm: at baseline, three times during the 12-week intervention program, and at 6 and 12-month follow-up visits.

**Table 1:** Schedule of enrolment and follow-up assessments of the RAPIDS Clinical Trial for the prevention of opioid overdose among people who use drugs

Activity	Time point (months)						
	Screen	Enrollment and post-allocation				Follow-up	
	−1	0	1	2	3	6	12
**Enrolment**							
Informed consent to screen	X						
Phone/email eligibility screen	X						
Informed consent to enroll		X					
Randomization allocation		X					
**Intervention**							
Motivational interview		X	X	X	X		
Role-play/teach-back		X	X	X	X		
Training video		X	X	X	X		
Usual care		X	X	X	X		
**Assessment**							
Self-report overdose						X	X
Urine drug screening		X	X	X	X	X	X
Sociodemographic characteristics		X	X	X	X	X	X
Substance use experiences		X	X	X	X	X	X
Overdose history		X	X	X	X	X	X
Fentanyl exposure							
Health care access		X	X	X	X	X	X
Physical and mental health		X	X	X	X	X	X
**Protocol deviation/adverse event reporting**	As needed throughout the protocol						

#### Sample size

Sample size/power calculations are based on findings from our pilot intervention study [14]. In this study, 37% of participants reported a lifetime history of overdose, and 10% reported a suspected fentanyl-related overdose in the past 6 months. Therefore, for the present study, we assumed that 20% of participants in the control arm will experience an overdose during the 12-month follow-up period. We conservatively assume an 80% retention rate in the trial, below the 90% retention rate observed in the pilot study. Given these assumptions, with a sample size of 500 participants, we have > 80% power to detect a 50% reduction in overdoses (i.e., the primary endpoint described above).

#### Recruitment and retention

As in our pilot intervention study, we will employ a combination of field-based recruitment techniques, Internet-based advertising, and state-wide public transport advertisement to recruit potential study participants. We will employ participant retention strategies that are effective at engaging PWUD, including dedicated study phones, comprehensive participant contact information tracking database, regular outreach in areas that participants are known to frequent, study visit honoraria (Brown University IRB-approved: $35 for baseline visit, $25 per follow-up visit), providing free parking to participants, and routinely searching the public Rhode Island Department of Corrections database to determine participant incarceration (considered lost to follow-up if incarcerated for greater than 90 days). For each visit, contact will be limited to a maximum of 10 attempts, after which participants will be considered lost to follow-up. If participants initiate contact after this, follow-up will continue according to the study schedule and contact attempts count reset.

### Assignment of interventions

#### Allocation and blinding

Eligible and consenting participants will be randomly assigned to either control or intervention arm using a simple 1:1 allocation using a permuted block randomization schedule, operationalized using the Randomization Module in REDCap™. Randomization will be assigned during the baseline visit following informed consent and completion of a baseline assessment (see below). Intervention allocations will be accessible in REDCap™ by authorized users only, such as the biostatistician, and revealed to interventionists only through REDCap™ by prompts during the baseline and follow-up visits.

### Data collection, management, and analysis

#### Data collection methods

##### Training for data collection staff

All staff will receive training for human subjects research. Additionally, staff will receive 8 h of relevant education (e.g., harm reduction, naloxone administration, overdose recognition and response, and stigma related to drug use) and 16 h of didactic and hands-on training in motivational interviewing processes and skills. The motivational interviewing component will be audio recorded and a subset reviewed for fidelity of implementation as an ongoing practice [33]. Prior to provision of the intervention, all interventionists will be trained in MI by a certified trainer, with random fidelity audits throughout the study period. Interventionists will undertake additional training where initial or audit assessments are below the 100% and 85% benchmarks, respectively.

##### Instruments and instrument-related procedures for data collection

Participant sociodemographics, substance use history, fentanyl knowledge, self-reported overdose and fentanyl exposure, and health service engagement will be captured in a structured biobehavioral questionnaire (Table 1). Standardized instruments will assess mental health (e.g., Generalized Anxiety Disorder 7-item scale; Center for Epidemiologic Studies Depression Scale; CDC Health-Related Quality of Life scale; and Addiction Severity-Index Lite) and substance use treatment (Subjective Opioid Withdrawal Scale; Opiate Dosage Adequacy Scale) outcomes. Urine specimens will be collected and tested for the presence of 12 substances using rapid qualitative test strips (Rapid Response™ Multi-Drug One Step Cup II, BTNX Corporation, ON, Canada): amphetamine (AMP-1S2-100; detection cut-off, 1000 ng/ml); benzodiazepine (BZO-1S3-100; detection cut-off, 300 ng/ml); buprenorphine (BUP-1S5-100; detection cut-off, 10 ng/ml); cocaine (COC-1S3-100; detection cut-off, 300 ng/ml); ethyl glucuronide (ETG-1S9-100; detection cut-off, 500 ng/ml); methadone (MTD-1S3-100; detection cut-off, 300 ng/ml); fentanyl (FYL-1S48-100; detection cut-off, 20 ng/ml); marijuana (THC-1S13-100; detection cut-off, 50 ng/ml); 3,4-methylenedioxymethamphetamine (MDM-1S-100; detection cut-off, 500 ng/ml); methamphetamine (MET-1S2-100; detection cut-off, 1000 ng/ml); morphine (MOP-1S3-100; detection cut-off, 300 ng/ml); oxycodone (OXY-1S27-100; detection cut-off, 100 ng/ml); and urine adulteration (URA-1S65).

#### Data management

##### Data management guidelines and procedures

The primary source of data will be survey responses, captured using REDCap™ software on laptops and tablet devices for direct, secure, and remote data entry. Probabilistic linkage of administrative datasets will provide an additional source of information regarding opioid overdose events for exploratory analyses. Ongoing surveillance efforts by the Rhode Island Department of Health captures data on all fatal overdose events occurring in the state through the Rhode Island Office of State Medical Examiners, and hospitalization and emergency medical service utilization related to suspected non-fatal opioid overdose, as described above [34]. Data will be transferred from the Rhode Island Department of Health to Brown University School of Public Health using *Health Insurance Portability and Accountability Act*-compliant secure file transfer protocols.

### Statistical methods

#### Primary outcome

Using the intention-to-treat procedure, we will determine the efficacy of the intervention by comparing self-reported rates of overdose among those assigned to the intervention arm (RAPIDS intervention) versus the control arm (usual care). A chi-square test of a two-by-two table (statistically significant at *p* < 0.05, two sided) will compare exposure to the intervention and the composite measure of self-reported overdose (defined as a “negative reaction from using too much drugs”) in the previous month at the 6- and/or 12-month follow-up visit.

#### Sensitivity analysis of primary outcome

We will repeat our analysis of the primary outcome using (1) pooled logistic regression to determine the independent effect of the intervention on self-reported overdose at either or both follow-up visits, adjusting for known or anticipated prognostic variables measured at baseline (e.g., injection drug use, history of overdose); (2) generalized linear mixed effects models (GLMM) with a logit link function to examine differences in self-reported overdose rates at 6 and 12-month visits; and (3) a per-protocol sub-analysis to determine the effect of the intervention on self-reported overdoses among those who used the fentanyl test strips compared to those who did not.

#### Secondary outcome

Using administrative-linked data of fatal and non-fatal overdose events, Kaplan-Meier estimators and Cox proportional hazards models will be used to determine the hazard of overdose events in the intervention and control groups. Right censoring for time-to-event analyses will occur at the time of first identified overdose, estimated date of loss to follow-up, non-opioid overdose-related mortality, or end of the study.

#### Missing data

At the completion of the study, patterns of outcome and covariate data missingness will be explored to determine the appropriate assumptions required for each affected variable. Using chained equations, the model will specify the conditional models for the missing outcome and covariates as missing completely at random (MCAR), missing at random (MAR), or missing not at random (MNAR). In the case of MNAR, we will specify a subset of the observations to derive the imputation model and may adjust imputed values by using specified shift and scale parameters for a set of selected observations in sensitivity analysis with a tipping-point approach. Finally, models with complete case, available data, and fully imputed data will be compared based on their coefficients, standard errors (SE), and *p* value for the covariates included in the model.

### Monitoring

#### Data monitoring

This study includes an independent Data and Safety Monitoring Board (DSMB) and a Data and Safety Monitoring Plan (DSMP). Further details on the DSMB charter are available on request. The DSMB will meet on a monthly basis for the first 6 months with the PI, Project Director, Biostatistician, and Study Coordinator to review the DSMP and protocol adherence. Thereafter, the DSMB will determine the schedule for future review meetings. The DSMB will also review any adverse or severe adverse events as outlined in the DSMP. Adverse events (including severe adverse events) will be captured using REDCap™ software during routine participant assessment and spontaneously as required, and reported to relevant parties as necessary.

Interim analyses will be performed when approximately one-third of participants are recruited to assess potential harms, and when approximately two-thirds of participants are recruited to assess efficacy. Using the alpha spending rule [35], *p* values will be constructed to maintain the overall study power of 0.05, two sided, assessing the proportion of expected events (i.e., self-reported non-fatal overdose) in each arm. If the test statistic exceeds the boundary, then the study could be considered for early termination due to emerging differences between the two arms. The study may be stopped due to (1) evidence of harm based on adverse and severe adverse event reporting and other safety data; (2) early evidence of efficacy from the second planned interim analysis; and (3) if any investigator judges it necessary for medical, safety, regulatory, or other reasons consistent with applicable laws, regulations, or good clinical practice. We do not propose that the trial be stopped for futility, as additional information collected at the 12-month follow-up may be useful for public health program planning and practice.

#### Auditing

The Principal Investigator is ultimately responsible for data safety and monitoring of the study. This process will be monitored on a weekly basis by his team, including the Project Director, Biostatistician, Study Coordinator, and Data Manager, with quarterly updates to the entire team of Co-Investigators. The project team will be responsible for ensuring that all policies and processes outlined in the Data Use Agreement (with Rhode Island Department of Health) are followed accordingly and that data are transferred and shared on the agreed-upon timeline.

### Ethics and dissemination

#### Research ethics approval

Ethical approval for this study has been obtained from the Brown University Institutional Review Board (ref: 1904002388) and other relevant Institutional Review Boards. Any modifications to the trial protocol (e.g., eligibility, potential benefit or harms, study objectives or design, study procedures) will be subject to approval by Brown University Institutional Review Board and reported to relevant parties as necessary. Administrative amendments resulting in minor changes will be subject to approval by the Brown University Human Research Protection Program.

#### Consent

Consent will be obtained at the start of pre-screening (verbal or written) for eligibility, and e-signed informed consent at the start of the baseline visit for eligible participants. Participants will be offered a copy of the e-signed informed consent form via email. All participants will be given detailed explanations of their rights as human subjects, including the purpose of the study, length of time for the interview process, study requirements, and risks and benefits of the interventions. At each in-person visit, research personnel will complete a brief verbal assessment using the “Alert & Oriented X4” procedure, where the participants’ awareness of person, time, place, and situation is assessed. Furthermore, all research personnel are trained to recognize and respond to an overdose, including calling for medical assistance, administering naloxone, and performing rescue breaths. On-call clinicians and local behavioral health services are available in the event of an emergency.

#### Confidentiality

All trial-related information will be securely stored at the study site, either in locked filing cabinets in secured areas (paper documents) or in secure Windows-based servers using two-factor authentication (electronic records).

#### Dissemination policy

This study has been registered on www.ClinicalTrials.gov, and all summaries of the results will be published on ClinicalTrials.gov by the Principal Investigator as soon as they become available. Trial results will be shared in peer-reviewed journals and presented at relevant local, regional, and national scientific meetings; conferences; and invited lectureships in a timely fashion. Publication authorship will follow the International Committee of Medical Journal Editors guidelines. All final peer-reviewed manuscripts from the trial will be submitted to PubMed Central digital archive.

## Discussion

To date, no randomized clinical trials have examined the efficacy of rapid fentanyl test strips in combination with behavior change intervention for the prevention of opioid overdose events. Our pilot study of young people at risk of fentanyl exposure demonstrated a high willingness to use fentanyl test strips as a harm reduction tool and the feasibility of a brief training module for non-expert use [14, 16]. Furthermore, positive test strip results were associated with drug risk behavior changes [16]. The current randomized clinical trial aims to demonstrate the application of theory-driven motivational interviewing sessions that improve participant self-efficacy in drug risk behavior reduction alongside fentanyl test strip distribution and brief training in their use.

The results from this randomized clinical trial will need to be considered in light of some limitations. Firstly, participants are being drawn from an unknown sample frame and may not be representative of the greater population of PWUD in Rhode Island or other geographic locations. However, our participant recruitment and retention plan is informed by pilot studies of PWUD in Rhode Island and will consist of numerous and diverse strategies for participant engagement [36]. Secondly, evaluating the effectiveness of a behavior change and fentanyl test strip intervention versus standard overdose education and naloxone distribution is complicated by the subjective nature of overdose events. Our measure of self-reported overdose captures participants’ perceived negative reactions of using drugs, in line with the literature [29, 30]. In addition, we will collect symptomatology of overdose events to better describe the severity of non-fatal overdose events experienced by participants. To address issues with subjective measures of exposure to fentanyl, we have incorporated urine drug testing—for 12 drugs including fentanyl—in the protocol and a 3-day recall period for exposure to substances. Finally, loss to follow-up is a key issue for studies of PWUD, compounded by the illicit nature of substance use. We will implement comprehensive participant retention strategies, including text message and email reminders, participant honoraria and subsidized parking, and targeted canvassing in spaces where PWUD are known to frequent. To address potential bias related to loss to follow-up, administrative data linkage—capturing fatal overdose events reported by the medical examiner, and non-fatal overdose events attended to by emergency medical services or presented at emergency departments—will be utilized in exploratory analyses.

This randomized clinical trial serves as the first such work to evaluate the efficacy of novel drug testing technology combined with a behavioral change component. If found to be effective, the RAPIDS intervention will provide the basis for an affordable program that can be feasibly implemented by harm reduction services for the prevention of opioid overdoses around the world. In light of IMF contamination of non-opioid substances, novel interventions are necessary to protect the health of people who use drugs.

## Trial status

Protocol version number and date: version 1.2; 19 March 2020

Expected date recruitment begins: 1 August 2020

Approximate date when recruitment will be completed: 31 August 2022

## Data Availability

The Principal Investigator (PI) will oversee the management of the final trial dataset with the Biostatistician and Data Manager. The PI will determine access to the final trial dataset in instances of formal requests for substudy analyses from co-investigators or collaborators.
